# Assessment of the Quality of Life in Children and Adolescents with Myopia from the City of Varna

**DOI:** 10.3390/jcm14134546

**Published:** 2025-06-26

**Authors:** Mariya Stoeva, Daliya Stefanova, Dobrin Boyadzhiev, Zornitsa Zlatarova, Binna Nencheva, Mladena Radeva

**Affiliations:** 1 Department of Eye Diseases and Vision Sciences, Faculty of Medicine, Paraskev Stoyanov Medical University, 9000 Varna, Bulgaria; mariya.stoeva@mu-varna.bg (M.S.); dobrinboyadzhiev@gmail.com (D.B.); zizlatarova@gmail.com (Z.Z.); bnenkova@gmail.com (B.N.); mladenaradeva@gmail.com (M.R.); 2 Department of Eye Diseases and Vision Sciences, University Specialized Eye Hospital, 9002 Varna, Bulgaria

**Keywords:** myopia, myopia control, quality of life, multifocal contact lenses, socially significant

## Abstract

**Background:** The World Health Organization defines myopia as a global epidemic. Its growing prevalence and the increasingly early age onset all raise a major concern for public health due to the elevated risk of loss and deterioration of visual function as a result of myopia-related ocular pathological complications. However, it remains unclear whether the vision-related quality of life of patients with myopia is the same as in healthy individuals. The aim of the present study is to assess the quality of life in children and adolescents with myopia between the ages of 8 and 16 years, who underwent observation at USBOBAL-Varna. **Methods:** This study prospectively included 190 patients with myopia between −1.00 and −5.50 D, corrected with different optical aids. After a thorough physical ocular examination and inquiry into the best visual acuity with and without distance correction, specially designed questionnaires were completed by the patients and their parents/guardians for the purpose of the study. The data from the questionnaires was statistically processed. The mean age of the patients in the study was 11.65 years, 101 were female and 89 were male. Of these, 83 wore monofocal glasses, 50 were monofocal and 47 were multifocal contact lenses, and 10 wore ortho-K lenses. **Results**: No significant difference in best corrected visual acuity (BCVA) was found among the three types of optical correction (*p*-value > 0.05). Cronbach’s alpha of the questionnaire for all 10 factors was higher than 0.6, indicating acceptable internal consistency. Significantly higher scores were reported for overall, near, and distance vision, symptoms, appearance, attitude, activities and hobbies, handling, and perception for soft contact lens wearers than for spectacle wearers (*p*-value < 0.05). Ortho-K wearers performed better than spectacle wearers in all aspects except for pronounced symptoms (*p* = 0.74). No significant difference was found between ortho-K wearers and soft contact lens wearers for any factor (*p* > 0.05). **Conclusions**: Patients wearing spectacles and with myopia above −5.00 D had the highest anxiety scores and lower quality of life among all myopic participants. The research on the quality of life in children with myopia with different refractive errors and optical correction devices is crucial for improving corrective devices and meeting the needs of patients.

## 1. Introduction

Myopia is a type of ametropia characterized by a more highly refractive ocular medium and/or a greater axial length of the ocular globe [1]. As a result, the parallel rays coming from objects at infinity focus in front of the retina, which leads to blurred visual acuity of objects at a distance [1]. Due to its high incidence on a global scale, the World Health Organization (WHO) classifies it among the most common causes of reversible blindness—uncorrected refractive errors occur in 123.7 million people [2]. According to the WHO, high myopia is myopia of −5 diopters (D) or more, associated with an increased risk of blindness due to the ocular morbidity that accompanies myopia, including degenerative changes in the macula, optic nerve, and peripheral retina, retinal detachment, myopic choroidal neovascularization, glaucoma, and cataract [3]. A study conducted by Holden et al. estimated that the incidence of myopia and high myopia will continue to increase worldwide, affecting 5 billion and 1 billion people by 2050, respectively, or become a so-called myopia pandemic [4]. The onset of myopia at an earlier age is a significant risk factor for the progression and occurrence of high myopia in the future [5]. Among the factors that play a role in the development of this type of ametropia are time spent outdoors, prolonged work at close range, prenatal factors and heredity, socioeconomic status, and urbanization [5]. The prevalence and incidence of myopia in children varies between regions and countries, with an estimated prevalence of 30.6% in European populations and furthermore increasing numbers [5]. Among the numerous methods for myopia control, atropine is an effective pharmacological agent, achieving 50–90% control, depending on its concentration. Although low concentrations of atropine (0.01% and 0.05%) have been identified as effective with minimal side effects, they are still associated with pronounced symptoms such as dry mouth, photophobia, poor near vision, glare, and allergic conjunctivitis [6].

Orthokeratology is another effective optical device that reduces changes in the axial length by approximately 50% per year [6,7]. Previous studies have shown that at least 6 months of usage of ortho-K lens is required in order for the treatment to be effective and carries a risk of significant complications, including corneal staining, papillary conjunctivitis, and microbial keratitis [6,7]. The cost, intensity of follow-up, and additional skills required by both eye care professionals and patients also pose known barriers [7].

Progressive, bifocal, and multifocal spectacle lenses and soft multifocal contact lenses are also prescribed as optical correction devices but do not appear to have a significant effect on controlling the underlying progression of myopia [6,8]. Spectacles or contact lenses with a defocusing design may be alternatives for individuals who cannot physiologically or financially tolerate other aids aimed at myopia control [6].

Currently, young individuals with myopia and their parents show a positive attitude toward various optical corrective devices, mainly due to their safety, despite the variability in therapeutic efficacy [6]. Various studies on the diverse means of control and correction have shown that steadily more children and parents are trying to find an alternative to glasses that is aesthetically acceptable and provides better control of myopia [9,10,11]. Others indicate that contact lens wearers tend to perceive themselves, perform in sports, and are accepted socially much better [12,13]. Often, eye specialists pay attention to the visual acuity, subjective symptoms, and objective signs without paying attention to the patient’s self-esteem, level of anxiety, relationships with peers, performance in school, sports, hobbies, and extracurricular activities. However, the latter are also important for achieving optimal patient cooperation, encouraging the doctor–patient relationship, and improving the quality of life of people with myopia. The aim of this study is to assess the quality of life in children and adolescents with myopia between the ages of 8 and 16, who underwent observation at USBOBAL-Varna.

## 2. Materials and Methods

This prospective study was conducted at the University Specialized Hospital-Varna between May 2023 and September 2024. After obtaining written informed consent from the parents/guardians of the patients with myopia, the study included children and adolescents who met the following criteria:

(1) Aged 8 to 16 years.
(2) Corrected myopia with optical aids, including monofocal glasses, mono- or multifocal soft contact lenses, and ortho-K lenses.
(3) Refractive error of −0.50 to −5.50 D and cylindrical refraction not more than 0.50 D.
(4) Monocular best-corrected visual acuity (BCVA) for both eyes no less than 0.00 logMAR.
(5) No history of ocular surgery or trauma.
(6) Absence of systemic diseases that could affect the quality of vision,
(7) No reported psychiatric disorders.
(8) Verbal patients.
(9) With parents/guardians who signed a declaration of informed consent.

After a thorough eye examination and inquiry of best corrected visual acuity, specially designed questionnaires were completed for the purpose of the study. Since we did not find any questionnaires in the Bulgarian language in the literature that would examine the quality of life of children and adolescents with myopia, we developed a three-component questionnaire for this purpose that would examine the quality of life in different aspects according to the patient and his/her parent/guardian and the level of anxiety among children with myopia. For its creation, we studied the Pediatric Refractive Error Profile (PREP) and the Screening for Emotional Disorders Associated with Childhood Anxiety (SCARED) [14,15]. PREP is a questionnaire specifically designed to quantify vision-related quality of life in children and adolescents with ametropia. SCARED is a 41-item anxiety screening tool widely used with children and adolescents aged 6–18 years in different countries [14,15]. A physician helped children and parents understand the items of the questionnaire.

Name: ______________________________________ Date: _______________
Best corrected visual acuity: ________________________________________
Directions:

Below is a list of statements that describe how people feel. Read each statement and decide whether or not these statements apply to you. Then, for each statement, fill in the number that corresponds to how much you agree with the statement and that describes your feelings over the past 3 months.

Category
Statement
Assessment

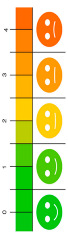

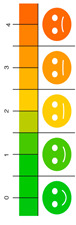
Attitude
I like wearing glasses/contact lenses.
HandlingI can easily clean and care for my glasses/contact lenses. My glasses/contact lenses are easy to put on and take off. My glasses/contact lenses get lost or break easily. My glasses/contact lenses fall off my face.

Overall vision
When I wear glasses/contact lenses, I have trouble seeing clearly. When I wear glasses/contact lenses, my vision is very clear. When I wear glasses/contact lenses, my vision is blurry.

Perception
When I wear glasses/contact lenses, my friends make fun of me. When I wear glasses/contact lenses, my friends want to wear glasses/contact lenses too. When I wear glasses/contact lenses, my friends like the way I look.

Symptoms
When I wear glasses/contact lenses, my eyes hurt. When I wear glasses/contact lenses, my nose, ears, or head hurt. When I wear glasses/contact lenses, my eyes itch, burn, or feel dry. When I wear glasses/contact lenses, my eyes feel good.

Near distance vision
When I wear glasses/contact lenses, I have no trouble seeing the computer screen or playing video games. When I wear glasses/contact lenses, I have troubles reading.

Appearance
When I wear glasses/contact lenses, I like the way I look. I don’t like how I look with glasses/contact lenses. If I wore contacts/glasses, I would look better.

Far distance vision
When I wear glasses/contact lenses, I can see clearly far away. When I wear glasses/contact lenses, I have trouble seeing movies or when looking far away.

School environment
When I wear glasses/contact lenses, I perform better in school. When I wear glasses/contact lenses, I do better on tests.

Activities and hobbies
I have never had a problem wearing glasses/contact lenses when playing outdoors. My glasses/contact lenses bother me when I play sports, dance, or do other activities.


Below is a list of statements that describe how people feel. Read each statement and decide if it applies to you. Then, for each statement, put a “✓” in the box that matches your answer.

0—Not True or Hardly Always True.

1—Somewhat True or Sometimes True.

2—Very True or Often True.

 Statement
 0  Not True or Hardly Always True

1

Somewhat True or Sometimes True
 2  Very True or Often True

1. I get headaches when I’m at school.




2. I worry about whether other people like me.




3. I am nervous.




4. I get stomachaches at school.




5. I worry about being as good as other children.




6. I’m worried about going to school.




7. When I get scared, I sweat a lot.




8. I find it difficult to talk to people I don’t know well.




9. I feel shy around people I don’t know well.




10. I am afraid to go to school.




11. When I get scared, I feel dizzy.




12. I feel nervous when I am with other children or adults and have to do something while they are watching me (for example: reading aloud, talking, playing a game, playing sports).




13. I feel nervous when I go to parties, dances, or other places where there will be people I don’t know well.




14. I am shy.

PART 2. (To be completed by parent)

Read the following questions about your child’s vision correction device and place a score from 0 to 4 in the column on the right.
*
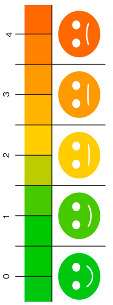
*
Category

Statement

Assessment

Attitude
Overall, how does your child feel about their optical correction device?

Handling
How does your child feel about their vision correction device (putting on/taking off, cleaning, fear of breakage, etc.)?

Overall vision

How does your child feel about their vision?


Perception
How does your child feel about their friends’ impressions of them because of their vision correction device?

Symptoms

How does your child feel about the comfort of their eyes?


Appearance
How does your child feel about their appearance?

School environment

How does your child cope in a school environment while wearing a corrective aid?


Activities and hobbies
How does your child feel about participating in activities while wearing vision correction?


For each question of the first panel, participants selected one response that best reflected their experience from “strongly agree,” “agree,” “neutral,” “disagree,” and “strongly disagree.” The statements were rated from 0 (most positive) to 4 (most negative) and then scaled from 0 (high quality) to 164 (low quality). The factor score was calculated as the average of all included questions.

The study was conducted in accordance with the Declaration of Helsinki, and the protocol was approved by the Ethics Committee of the Medical University of Varna №129 on 6 April 2023.

Statistical analysis of the data was performed using SPSS (IBM SPSS Statistics 20). The internal consistency was evaluated using Cronbach’s alpha. Normally distributed data were described as mean (MD) and standard deviation (SD), whereas non-normal distributed data were described as median. Normally distributed data were compared using the one-way analysis of variance, whereas non-normally distributed data were compared using the rank sum test. The prevalence of anxiety was compared using the chi-square test. A confidence interval (CI) of 95% was calculated to determine estimation precision. Statistical significance was set at *p* ≤ 0.05.

## 3. Results

The mean age of the patients in the study was 11.65 years (lowest age—8 years, highest—16 years). Of these, 89 were male and 101 were female. The demographic data of the patients included in the study are presented in Table 1.

Of the myopic subjects included in the study, 83 wore monofocal glasses, 50 were monofocal and 47 were multifocal contact lenses, and 10 were ortho-K lenses. No significant difference in best-corrected visual acuity (BCVA) was found among the three types of optical correction (*p*-value > 0.05) (Table 2).

Cronbach’s alpha of the PREP questionnaire for all 10 factors was higher than 0.6, indicating acceptable internal consistency (Table 3). Significantly better scores were reported for overall, near, and distance vision, symptoms, appearance, attitude, activities and hobbies, handling, and perception for soft contact lens wearers than for spectacle wearers (*p*-value < 0.05). Ortho-K wearers performed better than spectacle wearers in all aspects except for their pronounced symptoms (*p* = 0.81) (Table 4). No significant difference was found between ortho-K wearers and soft contact lens wearers for any factor (*p* > 0.05) (Table 4).

Cronbach’s alpha of the SCARED questionnaire for all factors was between 0.619 and 0.892, indicating acceptable internal consistency (Table 5). Significantly worse scores in the anxiety questionnaire were given by monofocal glasses wearers for symptoms, shyness, and performance than for contact lens wearers from all groups (*p*-value < 0.05).

**Figure 1:**
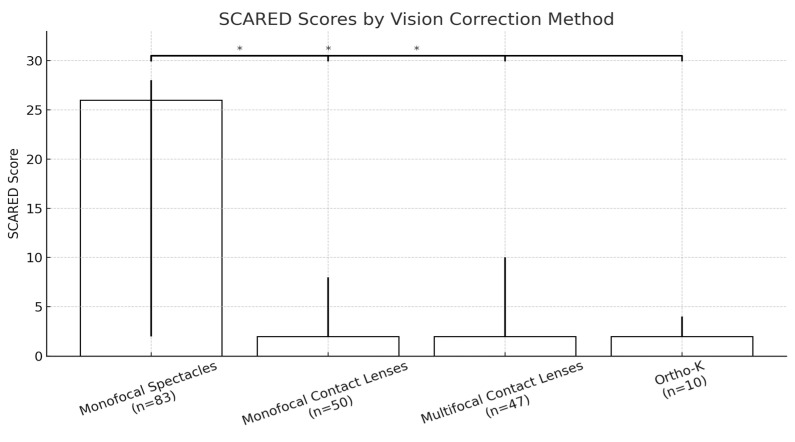
SCARED scores (median) of the participants in this study, distributed by corrective device. * indicates significant differences between groups.

**Figure 2:**
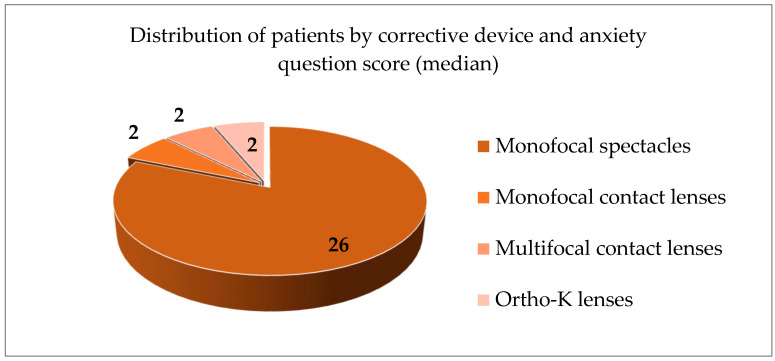
Distribution of patients based on the corrective device and the score of the anxiety questions panel (median).

In Figure 1, we report the SCARED scores, which indicated different mood or mental health disturbances among the participants in this study. Monofocal spectacle wearers had the highest anxiety scores among all the other participants—median = 26 (*p* < 0.05) as seen on Figure 2. There was no statistically significant difference observed between the monofocal and multifocal contact lens wearers and the ortho-k wearers—median = 2 for all groups (*p* > 0.05). Students with glasses performed poorer than monofocal contact lens wearers (26 (2, 28) vs. 2 (2, 8), *p* < 0.05), multifocal contact lens wearers (26 (2, 28) vs. 2 (2, 10), *p* < 0.05) and ortho-k wearers (26 (2, 28) vs. 2 (2, 4), *p* < 0.05).

In the following table, we have presented the results of the completed questionnaires distributed by categories according to the choice of refractive error correction means (Table 6). Quantitatively, we can once again observe that the monofocal spectacles group presented much higher scores overall compared to all other groups of patients.

**Figure 3:**
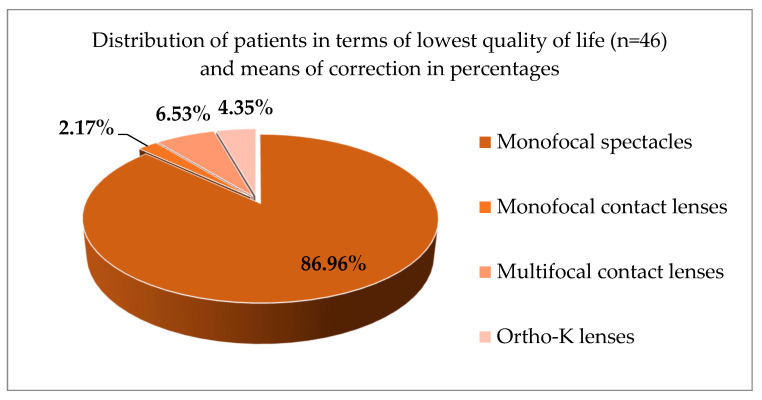
Distribution of patients in terms of lowest quality of life and means of correction in percentages.

According to the distribution of patients in terms of the lowest quality of life, presented by worst scores on the visual quality and the mood disturbance questionnaire, we noted a significantly higher percentage of participants (total n = 46) to be from the monofocal glasses group—86.95% of 46 patients that presented the lowest scores in terms of quality of vision and anxiety score (Figure 3). Lower quality of life, based on the questionnaire scores was calculated for each group, which was 24.21% for all participants (46 out of 190 patients). Forty patients were screened positive from the monofocal glasses group—86.96%; 3 from the multifocal contact lens wearers—6.53%; 2 from the ortho-K group—4.35%; and 1 from the monofocal contact lens wearers—2.17%. There was no significant difference between all contact lens wearers (*p* > 0.05).

**Figure 4:**
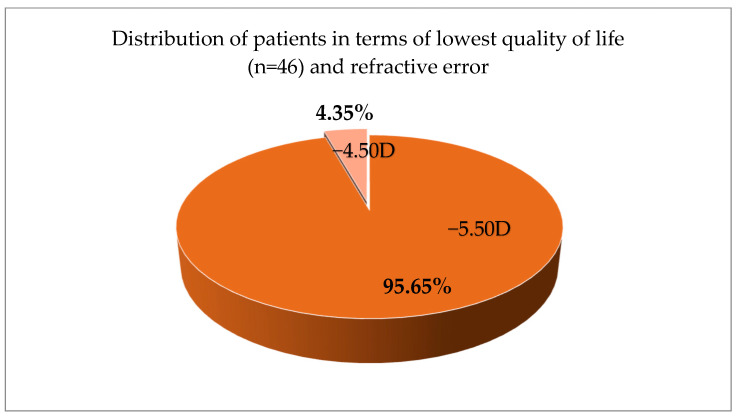
Distribution of patients according to the lowest quality of life and refractive error in percentage.

Patients with myopia above −5.00 D had the highest anxiety scores and lower quality of life among all participants with myopia—myopia of −5.50 D represents 95.65% of the total result of patients with lowest scores (n = 46) and −4.50 D for the other 4.35% (Figure 4). Only children and adolescents with myopia of −4.50 D and −5.50 D gave scores that classify them with a low visual quality or high anxiety score. This is why in Figure 3 we can only see those two refractive error ranges. No students with other refraction had a high anxiety and/or low visual quality score.

We did not find a statistically significant difference between the results obtained by parents and those provided by their children (*p* > 0.05).

## 4. Discussion

Myopia is a global public health problem with an increasing prevalence and incidence at earlier age onset and progression to a higher degree. Various optical and pharmacological interventions slow axial elongation in children. Their efficacy has been the subject of numerous studies, as have the quality of vision, the risk of adverse visual outcomes, overall safety, and the impact on vision-related quality of life [16,17,18,19].

Eye care professionals often measure the efficacy of myopia control clinically by examining the axial length, visual acuity, corneal condition, and anterior ocular surface [19]. However, patients’ subjective symptoms, attitudes, perceptions, and mental health also play a significant role in the success of a given therapy. Patient-reported outcomes with validated questionnaires allow for a quantitative assessment of the impact of a condition and its treatment and their impact on patients’ daily lives [20,21]. These results provide additional information to help assess the risk–benefit profile of a particular treatment for the individual patient (or group of patients) [20,21]. A study by Erickson et al. about the development and validation of a multidimensional scale for the quality of life of myopic patients involved creating a scale of 13 elements, associated with specific aspects of the frequency of visual compromise and ocular symptoms, with 13 corresponding elements for the level of tolerance to these issues: 3 elements related to cosmesis; 10 elements related to psychological characteristics; and 6 elements related to personality traits. The authors reported good internal consistency within each factor with Cronbach’s alpha range from 0.76 to 0.92. Zhang et al. also studied the quality of life of the myopic pediatric population using the PREP and SCARED questionnaires [19]. The authors reported good internal consistency values for 9 out of 10 factors on the PREP questionnaire [19]. Within our study, we have also disclosed similar findings—our internal consistency was in the range of 0.603–0.989 for all ten items. Furthermore, we notice a very impactful trend in new research on the topic of quality of life in myopic patients to include a variety of dimensions in order to be able to properly assess all aspects of life in which patients might be exhibiting deficiencies, discomforts, or a total absence [19,20,22,23,24,25].

However, limited studies have been conducted on this topic and even fewer have focused on adolescents and especially children. The present study demonstrates a relationship between the degree of myopia, the optical correction device, and the quality of life of patients. Walline et al. reported a higher Vision-Related Quality of Life (VRQoL) score in soft contact lens wearers in comparison to spectacle wearers in their studies from 2007 [26] and 2009 [12], especially in terms of physical appearance, athletic competence, academic performance, self-esteem, and social acceptance. Similar findings were reported by Rah et al. (2010) for activities, appearance, and satisfaction [27] and by Pomeda et al. (2018) for appearance, satisfaction, effect on activities, handling, peer perception, and overall score [18]. Our study also shows significantly higher scores for overall, near, and distance vision, symptoms, appearance, attitude, activities and hobbies, handling, and perception for soft contact lens wearers than for spectacle wearers (*p*-value < 0.05). When it comes to ortho-K lenses, a study conducted by Santodomingo-Rubido et al. reports higher VRQoL and acceptability with ortho-K in comparison to spectacle wearers in young children in Spain [28]. According to Zhao et al., ortho-K lenses improved the quality of life of the participants between ages 8 and 14 years in their study in terms of confidence and more time spent outdoors [29]. McAlinden et al. compared the quality of life of children and adults using different optical means including ortho-K, soft and gas-permeable contact lenses, and spectacles [30]. The research displays excellent psychometric properties, capable of quantifying VRQoL in patients with various forms of contact lenses [30]. According to our study, ortho-K wearers performed better than spectacle wearers in all aspects except for their pronounced symptoms (*p* = 0.81). We did not observe a statistically significant difference between ortho-K wearers and soft contact lens wearers for any factor (*p* > 0.05).

The negative assessment of the quality of life in our study was most strongly influenced by symptoms, handling, patients’ perception of their appearance, and the perceptions of their peers. As a result, we found that a large proportion of children who scored negatively in the latter two categories also showed greater anxiety about contacts with strangers and school attendance. In other studies, negative assessments were also found to be noted in the categories of symptoms, self and others’ acceptance, handling, and athletic performance [12,18,19,27,29].

In addition, patients with glasses and myopia greater than −5.00 D had the highest anxiety scores and lower quality of life among all myopic participants in our study. In other research conducted by Han et al., the more severe myopia resulted in a significantly reduced QoL score in both children and parents of children with any myopia [22]. Zawistowska et al. used the KIDSCREEN-27 questionnaire in adolescent myopia participants and reported that high myopia may have a negative impact on Health-Related Quality of Life (HRQoL), particularly affecting the physical and psychological well-being of girls [25]. The authors further elaborated on the importance of the holistic approach to high myopia treatment in such a young group of patients by taking into consideration their HRQoL scores as part of the diagnostic process [25].

In our study, children fitted with contact lenses showed significantly lower levels of anxiety, lack of difficulty in school, extracurricular activities, sports, and hobbies, and good overall self-acceptance and acceptance by peers. Children’s negative perception of their appearance may be associated with increased levels of stress, social anxiety, and feelings of shame and insecurity [31]. Dias et al. evaluated self-esteem and its relationship with various ocular and demographic characteristics in 469 myopic children participating in the Correction of Myopia Evaluation Trial (COMET) in 2002 [32]. Their multiple regression analyses showed that less symptomatic children in the study had higher self-esteem, but this also varied significantly by age, gender, and ethnicity (*p* < 0.05) [32]. The authors also reported that children who experience more visual symptoms (e.g., tired eyes or headaches) tend to evaluate themselves less favorably in terms of their physical appearance, academic performance and activities, and behavioral conduct [32]. In 2013, Dias et al. evaluated whether contact lens use was associated with self-esteem in myopic participants from the COMET study [33]. Contact lens wearers had higher social acceptance, athletic competence, and behavioral conduct scores at baseline compared to eyeglass users [33]. The authors’ findings suggest that self-esteem may influence the decision to wear contact lenses and that they are, in turn, associated with higher self-esteem in individuals [33]. Congdon et al. also elaborate on the social pressure on adolescents and younger children against spectacle wear [34]. The authors of the study identified data worldwide on these pressures in children across a range of contexts, such as being teased or discriminated against, and that parents are also sensitive to social pressures and hesitate to obtain spectacles for their children due to the stigma associated with them [34]. The mentioned studies come from countries with low incomes, such as Brazil, India, and Tanzania [34]. Another study from a high-income country showed that adults with high myopia express different aspects that affect their quality of life, such as psychological, cosmetic, practical, and financial factors, and their quality of life is similar compared to that of a patient with keratoconus [35]. In our neighboring country Turkey, Yeter et al. reported that wearing spectacles in adolescence seems to be related to self-esteem and social anxiety [36]. The authors suggest an appropriate approach and a thorough psychological evaluation of adolescents who wear spectacles [36]. In our research, myopic children and adolescents with high myopia and/or spectacle wearers displayed much lower overall, symptoms, appearance, attitude, activities and hobbies, and perception and anxiety scores (*p*-value < 0.05) than the other groups. All the contact lens wearer groups demonstrated better tolerance, acceptance, and attitudes to their optical correction and almost no anxiety or mood disturbance in regard to their refractive error corrective aid.

As a result, eye health professionals, together with parents and children, should work cooperatively in choosing the optimal effective means for optical correction or control of myopia that is safe and does not negatively affect the quality of life and mental health of children and adolescents.

The limitations of the study included data validation and statistical bias. The aim of the study was to measure the quality of life of myopic children and adolescents via questionnaires, which included three main points of interest—visual quality, mood disturbances, and parents’ attitudes. This wide variety of dimensions should be validated within a pilot study, which has not been performed before in our country to our knowledge. Furthermore, the number of patients involved in the study is relatively small (n = 190) and they all come from the same geographic region; moreover, the distribution of myopic children using different optic aids for correction or control of their refractive error varies immensely—for example, only 10 students accounted for the ortho-K group, whereas the monofocal spectacles group was represented by 83 pupils. The imbalance between groups leads to statistical spectrum bias, and conclusions are rather hard to form. Further investigation is needed in order to additionally validate and enhance the reliability of the results of this study.

## 5. Conclusions

The quality of life of children and adolescents with myopia is related to the degree of their refractive error and the optical correction means. The quantitatively proven results in terms of symptoms, handling, and negative perception of appearance given by the patients themselves or their peers within the monofocal spectacles group and the acceptable internal consistency of the questionnaire are valid reasons to conduct a pilot study, which would be the first of its kind in Bulgaria assessing the quality of life in myopic students corrected by different optical means, and to validate a questionnaire in our native language. This will help eye-care professionals to improve the quality of life of their patients and collect valuable epidemiological data. The benefits of contact correction in terms of efficacy and mental health, in comparable medical circumstances to spectacle correction, need to be established.

## Figures and Tables

**Table 1:** Demographic data of the patients included in the study.

Indicator	Distribution
Gender	101 f; 89 m
Age	Mean age—11.65, youngest—8, oldest—16
Refractive error	Myopia (from −1.00 to −5.50 D)

**Table 2:** Distribution of patients included in the study according to their optical correction device and best-corrected visual acuity (BCVA).

Indicator	Monofocal Spectacles	Monofocal Contact Lenses	Multifocal Contact Lenses	Ortho-K Lenses	x^2^/F	p
BCVA right eye, LogMAR	0.00	0.00	0.00	0.00	1.978	0.435
BCVA left eye, LogMAR	0.00	0.00	0.00	0.00	0.703	0.834

**Table 3:** Reliability of the factors included in the questionnaires (Cronbach’s alpha), distributed according to the correction factor (PREP questionnaire).

Category	The Cronbach’s Alpha			
	Monofocal Spectacles	Monofocal Contact Lenses	Multifocal Contact Lenses	Ortho-K Lenses
Attitude	0.798	0.732	0.623	0.785
Handling	0.743	0.712	0.789	0.812
Overall vision	0.815	0.765	0.719	0.832
Perception	0.892	0.609	0.612	0.645
Symptoms	0.840	0.756	0.731	0.774
Near distance vision	0.712	0.832	0.845	0.812
Appearance	0.895	0.604	0.603	0.645
Far distance vision	0.891	0.603	0.612	0.892
School environment	0.989	0.645	0.813	0.634
Activities and hobbies	0.882	0.621	0.671	0.872

**Table 4:** Statistical significance of the PREP scores of the myopic participants in the study, categorized by their different means of refractive correction—*p*-value.

Category	*p*-Value			
	Monofocal Spectacles vs. Monofocal Contact Lenses	Ortho-K vs. Monofocal Spectacles	Ortho-K vs. Monofocal Contact Lenses	Ortho-K vs. Multifocal Contact Lenses
Attitude	<0.001	<0.001	0.89	0.90
Handling	0.001	0.08	0.07	0.08
Overall vision	0.05	0.01	0.54	0.45
Perception	<0.001	<0.001	0.90	0.89
Symptoms	0.03	0.81	0.08	0.06
Near distance vision	0.05	0.05	0.05	0.05
Appearance	<0.001	0.001	0.43	0.70
Far distance vision	<0.001	<0.001	0.65	0.75
School environment	0.32	0.65	0.68	0.87
Activities and hobbies	<0.001	<0.001	0.64	0.73

**Table 5:** Reliability of the factors included in the questionnaires (Cronbach’s alpha), distributed according to the correction factor (SCARED questionnaire).

Category	The Cronbach’s Alpha			
	Monofocal Spectacles	Monofocal Contact Lenses	Multifocal Contact Lenses	Ortho-K Lenses
Symptoms (I get headaches when I’m at school; I get stomachaches at school.; When I get scared, I sweat a lot.; When I get scared, I feel dizzy.)	0.892	0.778	0.732	0.774
Shyness (I find it difficult to talk to people I don’t know well.; I am nervous.; I feel shy around people I don’t know well.; I feel nervous when I go to parties, dances, or other places where there will be people I don’t know well.; I am shy.)	0.854	0.692	0.619	0.702
Performance (I worry about whether other people like me.; I worry about being as good as other children.; I’m worried about going to school.; I am afraid to go to school.; I feel nervous when I am with other children or adults and have to do something while they are watching me (for example: reading aloud, talking, playing a game, playing sports).)	0.889	0.634	0.742	0.753

**Table 6:** Results of completed questionnaires distributed by categories.

0–164	Median				
	n = 190	Monofocal Spectacles (n = 83)	Monofocal Contact Lenses (n = 50)	Multifocal Contact Lenses (n = 47)	Ortho-K Lenses (n = 10)
Overall score	35	107	12	25	28
Attitude	2	4	0.5	1	1
Handling	7.5	8	2.5	3	4
Overall vision	2	2	0	0	1.5
Perception	4	9	1	1	0
Symptoms	2	2	2	2	2
Near distance vision	1	0	0	1	0
Appearance	2.5	7	0	0	0
Far distance vision	4	4	3.5	4	5
School environment	8	7	8	8	8
Activities and hobbies	4	4	3.5	4	3.5

## Data Availability

The original contributions presented in this study are included in the article. Further inquiries can be directed to the corresponding author.
